# Alcohol and Longevity

**Published:** 1896-02-15

**Authors:** 


### The Hospital

An Institutional Journal of

The Medical Sciences and Hospital Administration, with

Vol. XIX.—No. 490.] AN EXTRA NURSING SUPPLEMENT. [Feb. 15, 1896.

### Alcohol and Longevity.

It is a fact beyond dispute that British civilisation has for many years shown a falling death-rate. Because that has been so most of us have assumed that the population, as a whole, is longer-lived than formerly. We are now assured, however, on the authority of the United Kingdom Alliance, that that is not so, the real truth being that a certain proportion of our people live somewhat longer lives than formerly, whilst another proportion live shorter lives than their forefathers. The cause of this shortening of life, we are asked to believe, is alcohol.

Now alcohol is, or is not, the cause of the heightened death-rate among a portion of our population. If it can be proved that it is the cause, and the sole or even the main cause, then by all means let it be proved, and let all reasonable men and women lay the fact to heart. We cannot believe that a convincing demonstration of so startling a circumstance would fall on deaf ears in a reasonable and fairly-educated country like our own.

The death-rate has fallen greatly during the last half century among children and men and women under middle age. For example, in 1841-50 the death-rate among children of 10 to 15 years of age was 5·1; whereas in the period 1885-90 the rate among children of the same age was only 2·8. On the other hand, among men and women who have reached and passed middle life the death-rate has, it is said, considerably increased. In 1841-50 the rate among people of the ages 55-65 was 31·8, but among the same class in the period 1885-90 it had risen to 35·2.

So far we seem to be on the solid ground of fact. What we want to know is the why and wherefore of the the fact. Is alcohol at the root of the change for worse, or is something else responsible? Is there one cause mainly or entirely, or are there several causes in combination which produce so ugly a blot on the fair fame of our scientific civilisation? The United Kingdom Alliance, by the pen of its secretary, Mr. White, bids us look to alcohol as the chief or even the sole cause of the evil, and one principal reason is given for the contention. The consumption of alcohol per head of the population, Mr. White tells us, has not diminished, as we fondly supposed, but increased. In 1852 it was equivalent to 3·360 gallons of proof spirit per head, but in 1890 it had risen to 4·145 gallons. Now, though this is so, it is nevertheless the case that drinking has largely diminished among certain classes of the population, and entirely ceased among other classes. So that the increased consumption must be limited to a relatively small number of our people, and the actual extent of increase must be among that number relatively large. In a word, among people under middle age there has been a steady decrease in the quantity of alcohol consumed, and a steady and enormous growth of total abstinence. Among these the death-rate has steadily and markedly gone down during the whole of the past half century. On the other hand, since there has been a vastly increased consumption for the whole population, and since that consumption can only have increased among actual drinkers, and those drinkers are persons at or above middle age, it follows of necessity that they must have been responsible for the whole of the increased consumption. Corresponding with this there is, as we have already stated, a heightened death-rate. The conclusion of the whole argument is, that middle-aged and old persons drink much more alcohol than they used to drink, and die a great deal faster in consequence.

The *Times* makes some very pertinent criticisms upon all this. Where, it asks, does Mr. White get his statistics from; and how much ground do they actually cover? Their value for generalising, and, indeed, for any really useful purpose, must depend very largely upon the answers to these criticisms. As a matter of fact, the statistics cover no more than 422 cases, and are ten years old. On the general question of "alcohol and longevity" there is much to be said. Our own view of the matter is that, assuming for the moment a somewhat abbreviated longevity among our middle-aged and old population, alcohol is not the sole, or even the chief, cause. Even if it were, we should still be disposed to ask a question prior to all those raised by Mr. White, namely this: If it be actually true that the middle-aged and old among us drink more alcohol now than formerly, why do they drink it? Is there anything in our civilisation, in our methods of work, in the strain to which we are all subjected, that makes our people drink? We certainly do not believe that the modern man or woman drinks for the mere animal love of drink, or for the pleasure of feeling the stimulus and excitement which alcohol produces.

What we want to get at is the truth in this matter; and not merely that part of the truth which consists in the answers to the question, whether or not the middle-aged and old do drink more than formerly, or do die sooner in consequence; but whether or not there are causes and reasons inherent in our present civilisation and ways of life which predispose to the use of stimulants, and predispose so strongly as to make it impossible for men of average mental powers to resist the predisposition? In our judgment there are many very true, very real, and very obvious causes in our present ways of life which operate to produce profound depression of spirits in middle-aged and old men, and large impairments of the vital powers. But it is exactly in middle-age that the responsibilities of the civilised man are the heaviest. Those responsibilities he feels he must meet and face, just as the soldier on the battlefield feels that he has no choice but to meet and face even death if that be necessary. The real problem then we have to face is not this—Do middle-aged men drink alcohol and shorten their lives thereby? But this—If middle-aged men shorten their lives by drinking, why do they do it? and can we find any means of lessening the worry and strain of modern life, and so of preventing that depression of spirit and impairment of mental power which makes men feel a stimulant to be an imperative necessity?

